# The Effects of Second Primary Malignancies and Frailty on Overall Survival and Mortality in Geriatric Turkish Patients with Multiple Myeloma

**DOI:** 10.3390/curroncol30060423

**Published:** 2023-06-09

**Authors:** Yildiz Ipek, Nevra Karademir, Onur Yilmazer, Guven Yilmaz

**Affiliations:** 1 Department of Hematology, Kartal Lutfi Kirdar City Hospital, 34970 Istanbul, Turkey; cesus20@gmail.com; 2 Department of Internal Diseases, Kartal Lutfi Kirdar City Hospital, 34970 Istanbul, Turkey; nevrakarademir@gmail.com (N.K.); onur.ylmzr@gmail.com (O.Y.)

**Keywords:** multiple myeloma, frailty, second primary malignancy, overall survival

## Abstract

The study aims to investigate second primary malignancy (SPM) development and frailty in Turkish geriatric patients with multiple myeloma (MM) and to assess the relationship between overall survival (OS) and various characteristics including SPM and frailty. Seventy-two patients diagnosed with and treated for MM were enrolled in the study. Frailty was determined by the IMWG Frailty Score. Fifty-three participants (73.6%) were found to have clinically-relevant frailty. Seven patients (9.7%) had SPM. Median follow-up was 36.5 (22–48.5) months, and 17 patients died during the follow-up period. Overall (OS) was 49.40 (45.01–53.80) months. Shorter OS was found in patients with SPM (35.29 (19.66–50.91) months) compared to those without (51.05 (46.7–55.4) months) (Kaplan–Meier; *p* = 0.018). The multivariate cox proportional hazards model revealed that patients with SPM had 4.420-fold higher risk of death than those without (HR: 4.420, 95% CI: 1.371–14.246, *p* = 0.013). Higher ALT levels were also independently associated with mortality (*p* = 0.038). The prevalence of SPM and frailty was high in elderly patients with MM in our study. The development of SPM independently reduces survival in MM; however, frailty was not found to be independently associated with survival. Our results suggest the importance of individualized approaches in the management of patients with MM, particularly with regard to SPM development.

## 1. Introduction

Multiple myeloma (MM) is a hematological malignancy of plasma cells, which is characterized by monoclonal antibodies, which is particularly common in elderly patients [1]. The age-standardized global MM incidence is 1.78 per 100,000 people, and the global mortality rate was 1.14 per 100,000 people in 2020 [2]. While there is an increasing trend in the incidence of MM and especially in men, people aged 50 years and over, and those from high-income countries, the overall decreasing global trend in mortality was more evident in women [1]. Median survival times have improved over the past three decades, largely in parallel with the introduction of new therapeutic methods, such as autologous stem cell transplants (ASCT) in the early 1990s, immune modulatory agents (IMIDs) in the late 1990s, proteasome inhibitors in the early 2000s, second/third generation IMIDs, proteasome inhibitors, and others [3]. The effective biological mechanisms and decreased side effect profiles of these new therapies have made them mainstays of treatment in new-onset and relapsed MM. Despite improvements in outcomes, there is a disparity between younger and older MM patients, and fewer patients over 65 years of age benefit from novel therapies, possibly due to aging-related changes, co-morbidities, treatment-related toxicity, and drug discontinuation [4]. Increased frailty, which can be defined as vulnerability to stress factors due to a progressive decline in organ function, is among the factors of concern in this context [5]. The existence of frailty is taken into account in therapeutic decisions and in predicting the tolerability of the treatment, especially in the geriatric patient group who have a high prevalence of comorbid diseases [6]. Although the importance of frailty is increasingly recognized, it is not always easily identified without objective assessment.

With the prolongation of life expectancy among MM patients, the development of long-term comorbidities, including second primary malignancies (SPM)—both solid tumors and hematological cancers—has emerged as a serious issue in the management of MM [3]. Several clinical trials have suggested that MM patients treated with melphalan or IMIDs may have an elevated risk of developing SPM, with varying incidences among different ethnic groups [7,8]. This has caused some concern among treating physicians, MM patients, and policy makers. However, the incidence and precise mechanism of SPM development in the elderly MM population has not been well characterized so far.

The aim of the study was to determine the incidence of SPM and frailty in Turkish geriatric patients with MM, and to evaluate the potential relationships between overall survival (OS) and various factors, including clinical, biochemical, and therapeutic variables, as well as SPM and frailty.

## 2. Materials and Methods

### 2.1. Study Design

**Figure 1:**
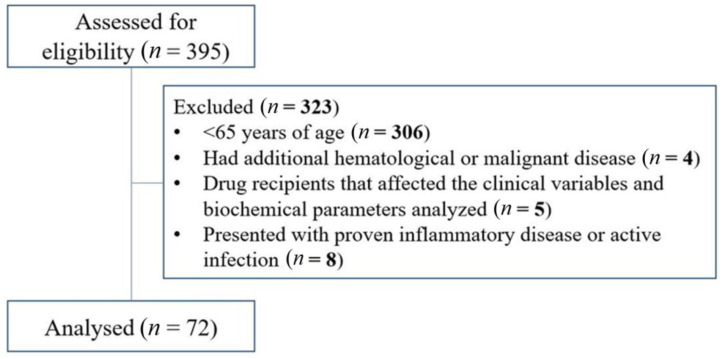
Flow diagram of the study.

This study was carried out retrospectively from 2017 to 2022 in the Department of Hematology, Kartal Dr. Lutfi Kirdar State Hospital, Istanbul, Turkey. The diagnosis of MM was performed according to the updated criteria defined by the 2014 International Myeloma Working Group (IMWG) and requires the presence of one or more myeloma-defining events (MDEs) in addition to evidence of either ≥10% clonal bone marrow plasma cells or a biopsy-proven bony or extramedullary plasmacytoma [9]. MDE involves evidence of end organ injury that can be attributed to the underlying plasma cell proliferative disorder, specifically established CRAB features (hypercalcemia, renal failure, anemia, or lytic bone lesions), and three specific biomarkers: clonal bone marrow plasma cells percentage of 60% or higher, serum free light chain (FLC) ratio of 100 or higher (provided involved FLC level is ≥100 mg/L), and more than one focal lesion on magnetic resonance imaging. The staging of the disease was determined by the revised MM International Staging System (R-ISS) [10]. R-ISS stage I was described as a serum β2-microglobulin level less than 3.5 mg/L and a serum albumin level ≥3.5 g/dL. R-ISS stage II included all patients with neither stage I nor stage III diseases. R-ISS stage III was defined as a serum β2-microglobulin level ≥5.5 mg/L, independent of the serum albumin level. Participants under the age of 65 years, those with history of concomitant hematological or malignant disorders, recipients of drugs that affect analyzed clinical variables and biochemical parameters, and patients with established inflammatory diseases or active infection were excluded. A total of 395 patients were selected for the study. Of these, 306 were excluded because they were under 65 years of age, 4 had additional hematological or malignant disease, 5 were drug recipients which affected the clinical variables and biochemical parameters analyzed, and 8 presented with proven inflammatory disease or active infection. A total of 72 patients diagnosed with and treated for MM were enrolled in the study (Figure 1).

All procedures performed in studies involving human participants were in accordance with the ethical standards of the institutional and/or national research committee and with the 1964 Helsinki Declaration and its later amendments or comparable ethical standards. The study was evaluated and approved by the Clinical Research Ethics Committee of Kartal Lutfi Kirdar City Hospital (Decision date: 11 January 2023, decision no: 2022/514/241/2).

### 2.2. Data Collection

Demographic and clinical variables, including age, sex, body mass index (BMI), type and staging of MM, comorbidities, such as chronic renal failure, congestive heart failure, hypertension, thyroid conditions, diabetes mellitus, neuropathy, deep vein thrombosis, renal and bone involvement, and the presence of SPM, frailty, anemia or hypercalcemia, as well as duration of follow-up, survival data, treatment agents, treatment response, ASCT data, cytogenetic results, and laboratory findings at diagnosis were obtained from patients’ medical files. Frailty was determined by the IMWG Frailty Score, which includes age, deficits in activities of daily living (ADLs), impairments in instrumental ADLs, and the Charlson Comorbidity Index [11]. BMI was calculated as body weight (kg) divided by height^2^ (m^2^).

### 2.3. Patient Management and Survival-Related Definitions

All participants had received 4 or 6 cycles of bortezomib, cyclophosphamide, and dexamethasone (VCD) for induction therapy. Since only VCD is reimbursed by the social security institution in our country, this regimen was used as the standard induction regimen in all patients. Maintenance therapy was applied to all patients who had at least partial response to ASCT. Forty-one patients underwent ASCT. Lenalidomide was administered after ASCT as maintenance treatment. Eight cycles of LEN/DEX (Lenalidomide–dexamethasone), KRD (Carfilzomib–lenalidomide–dexamethasone), VRD (Bortezomib-lenalidomide-dexamethasone), KD (Carfilzomib-dexamethasone), POM/DEX (Pomalidomide plus low-dose dexamethasone), or VEL/DEXA (Bortezomib–dexamethasone) were used for consolidation therapy. Consolidation therapy selection was based on the patient’s eligibility for ASCT, previous or current heart failure, Eastern Cooperative Oncology Group (ECOG) performance score, age, physical condition, fragility, response level to previous medications, relapse status, comorbidities, presence of kidney failure or neuropathy, oral or intravenous treatment, and social support. The responses to pre- and post ASCT, induction therapy, and consolidation therapy were determined according to IMWG response criteria [12]. Response were evaluated by whole-body computerized tomography, or whole-body magnetic resonance imaging or positron emission tomography in terms of the normalization of the bone marrow signal in previously affected areas, the decrease in the number and size of focal lesions, the resolution of severely infiltrated bone marrow infiltrate into focal lesions, and the decrease in the of number and size of soft tissue tumors. Palliative radiotherapy, zoledronic acid, or denosumab were used for skeletal-related events. All patients received standard supportive care measures, including blood transfusions and prophylactic or therapeutic antibiotics according to guidelines. OS was defined as the time from diagnosis to death due to any cause. Surviving and disease-free patients were censored at the last control visit.

### 2.4. Laboratory and Genetic Analyses

Blood samples were drawn from the antecubital vein after 12-h fasting on the day of hospital admission and were centrifuged at 5000 rpm (1500× *g*) for 10 min to separate the serum. Serum total protein, albumin, creatinine, urea, calcium, lactate dehydrogenase (LDH), alanine aminotransferase (ALT), aspartate aminotransferase (AST), gamma-glutamyl transferase (GGT), and urinary protein were measured with photometric methods on an Abbott Architect c8000 analyzer via the use of commercially available kits (Abbott Laboratories, Abbott Park, IL, USA). Estimated glomerular filtration rate (eGFR) was calculated by the equation based on serum creatinine, age, and sex. M-spike was determined using serum electrophoresis. Serum IgG, IgA, and IgM were measured via the nephelometric method on a Beckman IMMAGE system (Beckman Coulter, Indianapolis, IN, USA). Serum-free light chain (sFLC) kappa and lambda were determined by the monoclonal antibody-based nephelometric method on a Siemens BN system (Siemens Healthcare Diagnostics GmbH, Marburg, Germany), and the FLC ratio was calculated. Serum Beta-2 microglobulin was measured by the immunoturbidometric method on a Beckman AU680 analyzer (Beckman Coulter, Indianapolis, IN, USA). Cytogenetic analyses, including t(4;14), del(17p), del(13), t(11;14) and t(14;16), were performed using fluorescence in situ (FISH) in the same laboratory. High genetic risk was defined with IMWG criteria as the presence of monosomy 13 (−13) or del(13q), del(17p), t(4;14) or t(4;16) in the cytogenetic analysis of bone marrow samples, or when interphase FISH identified t(4;14), t(14;16), or del(17p) in MM cells [9].

### 2.5. Statistical Analysis

All analyses were performed on SPSS v25.0 (IBM Corp., Armonk, NY, USA). For the normality check, the Kolmogorov–Smirnov test was used. Normally-distributed continuous data are given as mean ± standard deviation, and non-normally distributed data are given as median (1st quartile–3rd quartile). Categorical data were summarized with count (*n*) and percentage. Relationships between SPM development and cytogenetic results were evaluated via Fisher’s exact test. Survival was analyzed with the Kaplan–Meier method and comparisons between groups were performed with the Log rank test. The multivariate cox proportional hazards model with forward conditional elimination of non-significant variables was performed to determine significant factors independently associated with mortality. Into the model, variables that were significant in the Log rank test and various continuous variables were included. Proportionality assumption in the multivariate cox proportional hazards model was checked by using the Schoenfeld residuals. The scatter plot of the residuals against time had −0.003 slope (*p* = 0.801) for SPM and 0.126 slope (*p* = 0.886) for ALT, meaning that both significant variables fulfilled the proportionality assumption. *p* values of <0.05 were accepted as being statistically significant.

## 3. Results

### 3.1. Descriptive & Inferential Analysis

Seventy-two patients with MM were included in the study. Demographic and clinical characteristics are summarized in Table 1. Median age of patients was 68 (66–72) years at the time of diagnosis, and 45 patients (62.5%) were male. Median diagnosis date was 23 May 2019 (16 October 2018–23 May 2020). The median follow-up period was 36.5 (22–48.5) months, and 17 patients died during follow-up. SPM was identified in 9.7% and frailty was identified in 73.6% of patients. Median SPM development time from diagnosis was 18 (14–22) months. SPM was significantly more frequent in patients who died, while there was no difference between survivors and deceased patients in terms of frailty.

Treatments are depicted in Table 2. All patients were treated with VCD as induction therapy; 64 received four cycles. Induction therapy resulted in complete response in 9 patients (12.5%), very good partial response in 35 patients (48.6%), partial response in 19 patients (26.4%), and stable disease in 9 patients (12.5%). Forty-one subjects (56.9%) underwent ASCT. In the evaluation of responses to consolidation therapy, complete response was observed in 16 (22.2%) subjects, very good partial response in 14 (19.4%) patients, partial response in 21 (29.2%), stable disease in 12 (16.7%), and progressive disease in 9 (12.5%) patients.

Biochemical characteristics are summarized in Table 3. A total of 26 patients (36.1%) presented with high serum FLC ratio (≥100),and 18 (25%) demonstrated high plasma cells in bone marrow (≥60%). A total of 33 subjects (46.5%) had proteinuria, and high genetic risk was defined in 19 (26.4%). We found no relationships between SPM development and the following: del17p (*p* = 0.583), t(4;14) (*p* = 0.527), t(14;16) (*p* = 1.000), and high genetic risk (*p* = 0.667).

### 3.2. Survival Analysis

**Figure 2:**
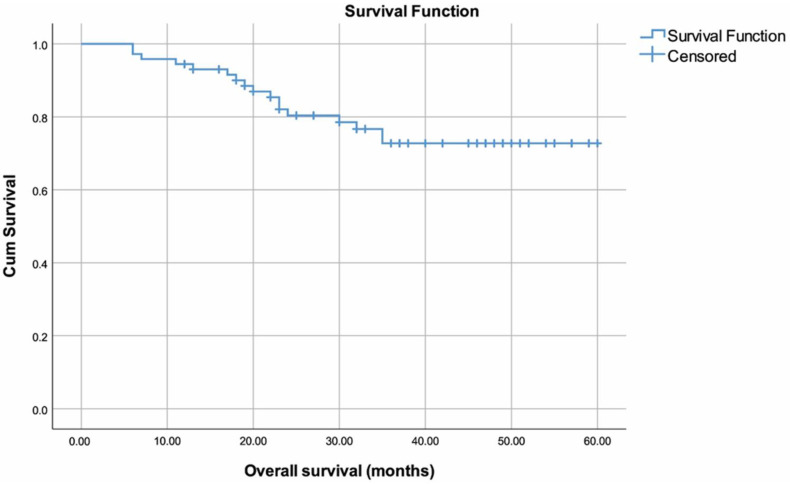
Overall survival plot.

**Figure 3:**
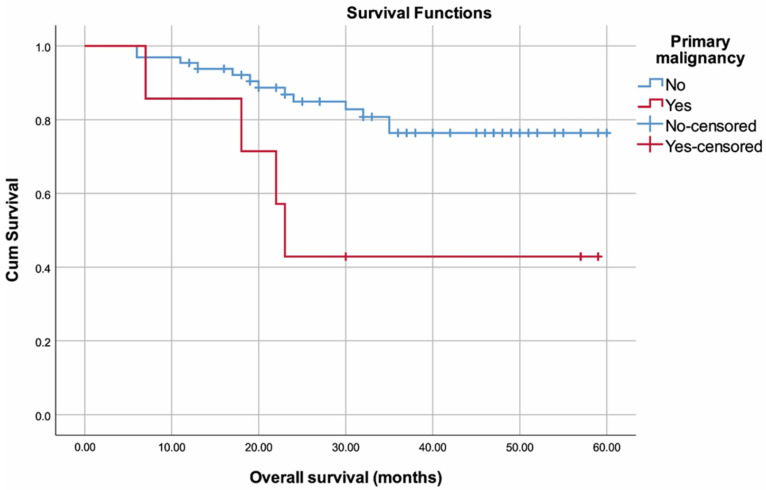
Overall survival plot with regard to second primary malignancy.

Survival-related data are summarized in (Table 4). Survival time was 49.40 (45.01–53.80) months overall (Figure 2). Lower OS rates were observed in patients with SPM (35.29 (19.66–50.91) months) compared to those without (51.05 (46.7–55.4) months) (*p* = 0.018) (Figure 3). No other variables were found to be significantly associated with OS (*p* > 0.05).

**Figure 4:**
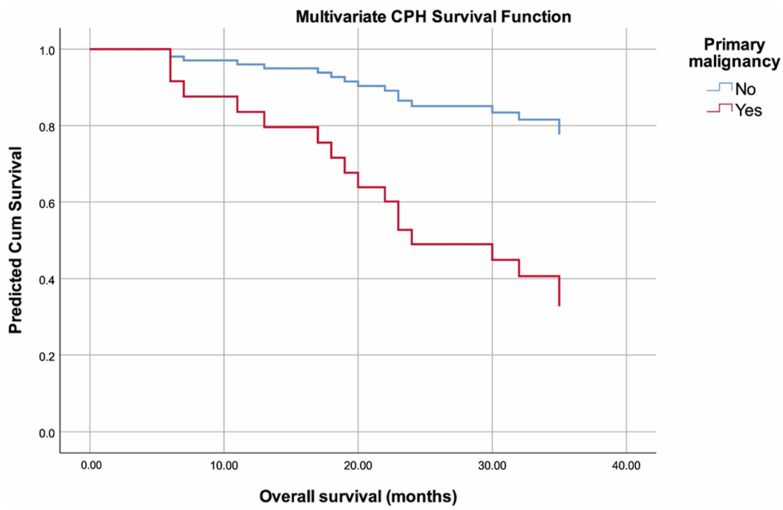
Predicted multivariate cox proportional hazards model plot with regard to second primary malignancy.

The multivariate cox proportional hazards model (forward conditional method) revealed that patients with SPM had 4.420-fold higher risk of death than those without SPM (HR: 4.420, 95% *CI*: 1.371–14.246, *p* = 0.013) (Figure 4). In addition, we found that high ALT level (*p* = 0.038) was independently associated with higher risk for mortality (Table 5). All other variables included in the analysis were non-significant, including age (*p* = 0.686), BMI (*p* = 0.534), M-spike (*p* = 0.282), IgG level (*p* = 0.793), IgA level (*p* = 0.646), IgM level (*p* = 0.809), sFLC kappa (*p* = 0.158), sFLC lambda (*p* = 0.956), sFLC kappa to lambda ratio (*p* = 0.143), serum total protein (*p* = 0.597), albumin (*p* = 0.572), creatinine (*p* = 0.109), eGFR (*p* = 0.076), calcium (*p* = 0.407), urea (*p* = 0.242), LDH (*p* = 0.880), Beta-2 microglobulin (*p* = 0.327), AST (*p* = 0.900), GGT (*p* = 0.219), duration of first remission (*p* = 0.786), and number of courses of consolidation therapy (*p* = 0.353).

## 4. Discussion

### 4.1. Summary and Contributions

This study aimed to investigate the incidence of SPM and frailty in Turkish geriatric patients with MM and to assess the relationship between OS and clinical, biochemical, and therapeutic variables, including SPM and frailty. We found the incidence of SPM to be 9.7% and frailty to be 73.6% in our study population. We demonstrated lower OS rates in patients with SPM compared to those without. We also showed that SPM presence and higher ALT were independently associated with mortality. Other clinical, biochemical, and therapeutic variables, including frailty, were not associated with OS or mortality.

MM is most commonly diagnosed in elderly patients, as evidenced by the fact that two-thirds of patients are aged over 70 years at diagnosis, and data from the United States shows that median age at diagnosis is 69 years [13]. Consistently, we found the median age at diagnosis to be 68 years in our study population. As life expectancy increases, the absolute burden of MM is expected to increase. It is estimated that by 2030, three out of every four new MM patients diagnosed will be over 64 years of age, notwithstanding various sex, racial, and ethnic variations [14]. These indicate the need for more effective and less-complicated MM treatments, especially for patients older than 65 years who have poor prognosis. Basic parameters including age, performance status, and comorbidity have traditionally been used in treatment selection for patients with MM [15]. In recent years, however, frailty indices have also received growing interest in the management of patients with hematological malignancies to achieve more appropriate personalized treatments [16]. Frailty is a condition characterized by reduced cognitive and physical reserves that can result in a decreased capability to withstand stressors, including cancer and its management [17]. Several tools have been developed to evaluate frailty [18]. IMWG established a frailty score in 2015 based on pooled analysis of 869 newly diagnosed MM patients enrolled in three randomized controlled trials and found that 3-year OS was 57% in frail MM patients [11]. They showed that the IMWG-frailty score could predict survival and toxicity and was predictive for elevated risk of mortality, progression, treatment discontinuation, and non-hematological adverse events. Mina et al. demonstrated, with data from the EMN10-Unito study, that 25.1% of patients had frailty and reduced OS. Moreover, adverse events, dose modifications, and therapy discontinuation were more frequent in frail patients [19]. However, they showed similar overall response rates and very good partial response and progression-free survival, irrespective of frailty status [19]. Stege et al. reported in a study of 65 frail patients with newly diagnosed MM that the 12-month OS in the study population was 78% and median progression-free survival was 13.8 months [20]. Stege et al. also showed in another study of 220 newly diagnosed non-transplant MM patients (treated with dose-adjusted melphalan-prednisolone-bortezomib) that 61% of patients were frail, and frail patients had significantly inferior OS, more co-morbidities, and lower physical and cognitive function as compared to non-frail patients [21]. In a randomized, controlled, multicenter phase II study of 112 relapsed or refractory MM patients, Auner et al. found that the prevalence of frailty in the study population was 74%; however, no significant differences were found between frailty groups with regard to PFS and OS [22]. We used the IMWG-frail scoring system for our study population and found that 73.6% of patients were classified as frail. We did not find any relationships between frailty and analyzed outcomes (OS and mortality). The inconsistencies between our study and some previous studies may have been due to the wide heterogeneity in both the definition, categorization, and cut-off points used for frailty. In addition, the relatively small sample size of our study and the fact that it consisted of only one ethnic group with different treatment protocols may have affected our results. Further large-scale prospective studies with more sophisticated and standardized assessments of frailty are required to establish the relationship between the presence and management of frailty and the clinical outcomes of MM.

SPMs have been reported in patients diagnosed with MM since the 1960s [23]. Owing to advances in treatments and improved survival, SPMs are becoming a wide-spread reality in MM survivors. The incidence of SPM in MM patients has been estimated from population-based registry studies, prospective clinical studies, and retrospective analyses. Results range from 1% to 15%, but it is difficult to generalize the results of these studies due to ethnic differences, variations in treatment regimens over time, and heterogeneity of the studies [24]. We found the incidence of SPM to be 9.7% in our study population. This indicates that the increasing incidence of SPM is a crucial issue for MM management because of their potential to alter the treatment algorithm as well as change the prognosis. Palumbo et al. reported in a meta-analysis of 3254 newly diagnosed MM patients that SPM at 5 years had been identified in 6.9% of patients treated with lenalidomide as first line therapy and in 4.8% of those without this treatment [25]. McCarthy et al. showed in a meta-analysis of 1208 MM patients that the incidence of both hematological and solid SPMs were higher with lenalidomide in comparison to placebo or control [26]. These results suggest that there may be a relationship between the increased incidence of SPM in our study population and lenalidomide, which we routinely use as a maintenance therapy. Jonsdottir et al. demonstrated in a Swedish-based study of 26,627 patients diagnosed with MM between 1958 and 2011 that 1547 (5.8%) developed SPMs and that patients with SPMs had a 2.3-fold elevated risk of mortality compared to MM patients without SPM [27]. They also showed a median survival of 1.1 years after diagnosis of SPM, shorter than the 3-year survival period among non-SPM patients after the corresponding date. Turgutkaya et al. showed five (1.6%) SPM cases among 310 symptomatic Turkish MM patients, and these patients had shorter survival [7]. Wang et al. collected data from the SEER 9 Registry Database (1973–2018) on 43,825 newly diagnosed MM patients and found that a total of 3101 (7.1%) patients developed 3407 SPMs [28]. They also showed that age, race, sex, and time of diagnosis were significant factors for the risk of developing SPM, and that patients with SPM had a 1.4-fold higher risk of mortality. Consistently, we demonstrated lower OS rates in patients with SPM compared to those without SPM. We also showed that the presence of SPM was independently related to mortality, and patients with SPM had a 4.42-fold higher risk of mortality compared to those without SPMs. Our results support the hypothesis that SPM negatively impacts survival in patients with MM. Poor survival in MM patients with SPM may be caused by patient-related factors such as advanced age, male sex, race/ethnicity, genetic predisposition, co-morbidities, and disease-related factors (M-protein, immunoglobulin subtype, clonal hematopoiesis, myeloma genetics and immune dysregulation) [3,8]. Extrinsic risk factors may involve treatment protocols, as well as environmental and life-style factors known to advance cancer risk, such as smoking and obesity [3,8]. These risk factors, interacting or combined, may have contributed to the development of SPM and poor OS in our subjects. The importance of individualized, patient-specific, and integrated approaches should be considered in order to minimize SPM risk, improve quality of life, and increase OS in MM patients. In addition, it is crucial to detect the development of SPM; thus, patients may benefit from age-appropriate oncological screening.

Liver function can be routinely evaluated in clinical practice, as liver function tests, such as ALT and AST, are relatively sensitive indicators of hepatocellular damage. ALT catalyzes the transfer of amino groups to form products in gluconeogenesis and amino acid metabolism. Trauma, hypoxia, and cell membrane disruption and dysfunction can lead to increased mitochondrial swelling, cell permeability, and rupture, resulting in the release of AST and ALT into the bloodstream [29]. In conclusion, serum ALT and AST levels are jointly associated with the severity of liver damage. Various prospective reports have shown relationships between serum ALT levels and the risk of cardiovascular diseases, type 2 diabetes mellitus, vascular and nonvascular mortality, and all-cause mortality outcomes [30]. Malignant cells, as well as normal cells, can produce ALT, and it is increasingly recognized that ALT plays a significant role in tumorigenesis. Studies have reported that an elevated serum ALT level is significantly related with an adverse prognosis in various cancers, including cholangiocarcinoma, cancers in respiratory and intrathoracic organs, breast cancer, and hepatocellular and renal cell carcinoma [30,31]. However, the prognostic value of serum ALT levels in patients with MM is still unclear. A review of 869 MM cases followed from 1960 to 1971 at the Mayo Clinic found a palpable liver in 21% of the initial findings [32]. Poudel et al. showed that abnormalities in liver function were characteristic of MM, and serum ALT was elevated in 24 (64.9%) of 37 cases of MM [33]. Walz-Mattmuller et al. showed liver infiltration in 32% of MM samples in their study, in which they histologically and immunohistochemically examined the incidence and pattern of liver involvement in liver samples taken from 25 MM cases [34]. Kiba et al. demonstrated that patients with MM with high AST had worse prognosis than patients with low AST [35]. This is the first study to show the prognostic value of circulating ALT levels in MM patients. We found ALT levels to be one of the factors significantly associated with OS in MM patients, suggesting that patients with high ALT levels may have a worse prognosis than patients with low levels of this parameter. Although the mechanisms underlying the observed relationship are not completely clear, we think that lifestyle factors such as alcohol consumption, smoking, exercise, obesity, insulin resistance, and diet may be associated with the change in serum ALT levels and may contribute to OS. In addition, cancer cells exhibit a higher rate of aerobic glycolysis to produce more energy to support their high proliferation compared to normal cells. Glutaminolysis and pyruvate production are catalyzed by ALT, which is enhanced in tumor cells [36]. Thus, serum ALT levels may reflect the metabolic state in cancers, possibly related to tumor growth and progression. Furthermore, oxidative stress and inflammation are closely associated with cancer development, and high AST or ALT levels may represent the high-oxidative-stress and inflammatory environment in vivo associated with lower survival rates for patients with different types of cancer [37]. The results observed in our study may correlate with these mechanisms. Further studies are needed to examine the underlying biological mechanisms of ALT’s role in MM development and progression.

### 4.2. Strengths and Limitations

In addition to those noted before, the current study has some limitations, such as its cross-sectional and single-centered design, the relatively small number of patients, heterogeneity of treatment among subjects, and the limited clinical data available before onset of MM and SPM. We did not include patients younger than 65 years of age in our study. This created a study population comprised of elderly patients with a higher risk of comorbidity, and may have contributed to the results and could limit interpretations in terms of external validity. Our results may not be generalizable to other populations or age groups as we only provide data for Turkish MM patients older than 65 years.

### 4.3. Future Work

We plan to conduct a similar multicenter study consisting of different age groups, and further prospective studies that utilize longer follow-up periods and include more patients that account for patient-, disease-, and treatment-related factors. These measures will help validate our current data on frailty, SPMs, and survival in patients with MM.

## 5. Conclusions

We demonstrated high prevalence of SPM and frailty in elderly patients diagnosed with MM. The presence of SPM was found to be directly associated with increased mortality and worse OS. We also revealed that MM patients with high ALT levels may have a worse prognosis than patients with low levels of this parameter.

## Figures and Tables

**Table 1:** Clinical and demographic characteristics of participants.

Variables	Results
Age, years, (*n* = 72)	68 (66–72)
Sex, (*n* = 72)	
Male	45 (62.5%)
Female	27 (37.5%)
Body mass index, kg/m^2^, (*n* = 72)	25.40 ± 3.49
Type of MM, (*n* = 72)	
IgG kappa, (*n*, %)	27 (37.5%)
IgG lambda, (*n*, %)	13 (18.1%)
IgA kappa, (*n*, %)	17 (23.6%)
IgA lambda, (*n*, %)	8 (11.1%)
Kappa light chain, (*n*, %)	3 (4.2%)
Lambda light chain, (*n*, %)	4 (5.6%)
R-ISS, (*n* = 72)	
Stage II, (*n*, %)	19 (26.4%)
Stage III, (*n*, %)	53 (73.6%)
Co-morbidities	
Chronic renal failure, (*n*, %)	17 (23.6%)
Congestive heart failure, (*n*, %)	13 (18.1%)
Hypertension, (*n*, %)	44 (61.1%)
Thyroid conditions, (*n*, %)	10 (13.9%)
Diabetes mellitus, (*n*, %)	26 (36.1%)
Neuropathy, (*n*, %)	3 (4.2%)
Deep vein thrombosis, (*n*, %)	3 (4.2%)
Second Primary malignancy, (*n*, %)	7 (9.7%)
Renal involvement, (*n*, %)	21 (29.2%)
Bone involvement by FDG-PET, (*n*, %)	54 (75.0%)
Plasmacytoma, (*n*,%)	19 (26.4%)
The presence of frailty, (*n*, %)	53 (73.6%)
The presence of hypercalcemia, (*n*, %)	8 (11.1%)
The presence of anemia, (*n*, %)	63 (87.5%)
Follow-up time, months, (*n* = 72)	36.5 (22.0–48.5)
Final status	
Alive, (*n*, %)	55 (76.4%)
Exitus, (*n*, %)	17 (23.6%)

Data are given as mean ± standard deviation or median (first quartile–third quartile) for continuous variables according to normality of distribution and as frequency (percentage) for categorical variables.

**Table 2:** Treatment modalities of patients.

Variables	*n* (%)
Induction Therapy	
VCD, (*n*, %)	72 (100.0%)
Number of course, Induction therapy	
4, (*n*, %)	64 (88.9%)
6, (*n*, %)	8 (11.1%)
Response to induction therapy, (*n* = 72)	
Complete response, (*n*, %)	9 (12.5%)
Very good partial response, (*n*, %)	35 (48.6%)
Partial response, (*n*, %)	19 (26.4%)
Stable disease, (*n*, %)	9 (12.5%)
Progressive disease, (*n*, %)	0 (0.0%)
Autologous stem cell transplant, (*n*, %)	41 (56.9%)
Response of prior to ASCT, (*n* = 41)	
Complete response	5 (12.2%)
Very good partial response	25 (61.0%)
Partial response	11 (26.8%)
Response to ASCT, After 100 days, (*n* = 41)	
Complete response, (*n*, %)	25 (61.0%)
Very good partial response, (*n*, %)	15 (36.6%)
Partial response, (*n*, %)	1 (2.4%)
Maintenance treatment after ASCT, (*n* = 72)	
None, (*n*, %)	12 (16.7%)
Lenalidomide, (*n*, %)	60 (83.3%)
Consolidation Therapy, (*n* = 72)	
LEN/DEX, (*n*, %)	18 (25.0%)
KRD, (*n*, %)	8 (11.1%)
VRD, (*n*, %)	9 (12.5%)
KD, (*n*, %)	13 (18.1%)
POM/DEX, (*n*, %)	16 (22.2%)
VEL/DEXA, (*n*, %)	8 (11.1%)
Number of course, Consolidation Therapy, (*n* = 72)	8 (6–12)
Response to Consolidation Therapy, (*n* = 72)	
Complete response, (*n*, %)	16 (22.2%)
Very good partial response, (*n*, %)	14 (19.4%)
Partial response, (*n*, %)	21 (29.2%)
Stable disease, (*n*, %)	12 (16.7%)
Progressive disease, (*n*, %)	9 (12.5%)
Requiring Radiotherapy, (*n*, %)	17 (23.6%)
Requiring Zoledronic acid use, (*n*, %)	52 (72.2%)
Requiring Denosumab use, (*n*, %)	15 (20.8%)

VCD: Bortezomib–cyclophosphamide–dexamethasone therapy, ASCT: Autologous stem cell transplant, R-ISS: Revised International Staging System; LEN/DEX: Lenalidomide–dexamethasone, KRD: Carfilzomib–lenalidomide–dexamethasone; VRD: Bortezomib–lenalidomide–dexamethasone; KD: Carfilzomib–dexamethasone; POM/DEX: Pomalidomide plus low-dose dexamethasone; VEL/DEXA: Bortezomib–dexamethasone. Data are given as mean ± standard deviation or median (first quartile–third quartile) for continuous variables according to normality of distribution and as frequency (percentage) for categorical variables.

**Table 3:** Laboratory and genetic characteristics of participants.

Variables	Results
IgG, g/L	17.51 (6.97–28.81)
IgA, g/L	2.21 (0.54–9.33)
IgM, g/L	0.36 (0.23–0.49)
sFLC Kappa, mg/L	153 (55–296)
sFLC Lambda, mg/L	38.0 (22.5–163.5)
sFLC Kappa to Lambda ratio	4.75 (0.72–8.09)
M-Spike, g/dL	2.39 ± 1.13
Beta-2 microglobulin, mg/L	3.25 (2.57–4.95)
High plasma cells in bone marrow (≥60%), (*n*,%)	18 (25.0%)
High serum free light chain ratio (≥100), (*n*,%)	26 (36.1%)
Total Protein, g/dL	8.88 ± 1.78
Albumin, g/dL	3.41 ± 0.71
Creatinine, mg/dL	1.34 (0.93–2.40)
eGFR, mL/min/1.73 m^2^	52 (28–77.5)
Calcium, mg/dL	9.67 ± 0.93
Urea, mg/dL	42 (32–70)
LDH, IU/L	172.79 ± 45.81
High LDH (>248), (*n*,%)	4 (5.6%)
ALT, IU/L	14 (10–24.5)
AST, IU/L	19 (15–24)
GGT, IU/L	31 (19–58)
Urinary Protein, mg	225 (153–1064)
Proteinuria (*n*,%)	33 (46.5%)
Cytogenetic results	
del17p, (*n*,%)	10 (13.9%)
t(4;14), (*n*,%)	7 (9.7%)
t(14;16), (*n*,%)	2 (2.8%)
Other, (*n*,%)	24 (33.3%)
High genetic risk, (*n*,%)	19 (26.4%)

eGFR: estimated glomerular filtration rate, LDH: Lactate dehydrogenase; ALT: Alanine aminotransferase; AST: Aspartate aminotransferase; GGT: Gamma-glutamyl transferase. Data are given as mean ± standard deviation or median (first quartile–third quartile) for continuous variables according to normality of distribution, and as frequency (percentage) for categorical variables.

**Table 4:** Survival times (months) and comparisons of groups.

	*n*	Exitus	Mean (95% CI)	*p*
Overall survival	72	17	49.40 (45.01–53.80)	N/A
Second Primary malignancy				
No	65	13	51.05 (46.70–55.40)	0.018
Yes	7	4	35.29 (19.66–50.91)	
Frailty				
No	19	4	49.73 (40.92–58.53)	0.908
Yes	53	13	48.49 (43.51–53.48)	
Sex				
Male	45	8	52.07 (47.14–57.00)	0.163
Female	27	9	44.63 (36.78–52.47)	
Type of heavy protein chain				
IgG	40	8	50.26 (44.28–56.23)	0.959
IgA	25	6	50.05 (43.65–56.44)	
Type of light protein chain				
Kappa	47	12	48.16 (42.42–53.90)	0.473
Lambda	25	5	51.03 (44.67–57.39)	
Chronic renal failure				
No	55	13	49.48 (44.50–54.47)	0.975
Yes	17	4	48.64 (39.71–57.57)	
Congestive heart failure				
No	59	14	49.67 (44.93–54.40)	0.847
Yes	13	3	48.21 (37.50–58.92)	
Hypertension				
No	28	4	53.40 (47.51–59.28)	0.169
Yes	44	13	46.19 (40.28–52.09)	
Thyroid conditions				
No	62	17	47.71 (42.75–52.67)	0.073
Yes	10	0	(1)	
Diabetes mellitus				
No	46	8	52.65 (48.05–57.26)	0.070
Yes	26	9	43.16 (34.76–51.55)	
Neuropathy				
No	69	17	48.92 (44.36–53.48)	0.344
Yes	3	0	(1)	
Deep vein thrombosis				
No	69	16	49.65 (45.20–54.11)	0.534
Yes	3	1	24.00 (22.61–25.39)	
Hypercalcemia				
No	64	16	48.64 (43.83–53.44)	0.373
Yes	8	1	49.57 (41.54–57.61)	
Renal involvement				
No	51	13	47.90 (42.26–53.54)	0.361
Yes	21	4	52.36 (46.43–58.28)	
Anemia				
No	9	0	(1)	0.090
Yes	63	17	47.87 (42.96–52.78)	
Bone involvement by FDG-PET				
No	18	6	46.16 (37.37–54.95)	0.440
Yes	54	11	50.52 (45.55–55.49)	
High plasma cells in bone marrow (≥60%)				
No	54	14	47.51 (42.33–52.69)	0.412
Yes	18	3	52.83 (45.49–60.18)	
High serum free light chain ratio (≥100)				
No	46	13	47.17 (41.27–53.07)	0.200
Yes	26	4	52.66 (46.94–58.37)	
Plasmacytoma				
No	53	10	51.84 (47.28–56.40)	0.069
Yes	19	7	39.08 (30.29–47.86)	
LDH				
Normal	68	17	48.67 (44.03–53.32)	0.241
High	4	0	(1)	
Proteinuria				
No	38	8	50.39 (44.49–56.28)	0.663
Yes	33	9	47.75 (41.37–54.13)	
Genetic risk				
Low	53	14	47.63 (42.15–53.12)	0.254
High	19	3	49.00 (42.80–55.20)	
Frailty				
No	19	4	49.73 (40.92–58.53)	0.908
Yes	53	13	48.49 (43.51–53.48)	
Requiring Radiotherapy				
No	55	13	49.69 (44.81–54.56)	0.920
Yes	17	4	44.35 (35.94–52.77)	
Requiring Zoledronic acid use				
No	20	3	52.72 (46.21–59.24)	0.232
Yes	52	14	47.60 (42.08–53.11)	
Requiring Denosumab use				
No	57	15	48.06 (42.89–53.23)	0.270
Yes	15	2	53.36 (46.13–60.59)	
Number of course, Induction Therapy				
4	64	17	47.14 (42.35–51.94)	0.084
6	8	0	(1)	
Response to Induction Therapy				
Complete response	9	2	54.44 (47.65–61.24)	0.334
Very good partial response	35	8	34.95 (30.67–39.23)	
Partial response	19	3	49.68 (44.14–55.23)	
Stable disease	9	4	34.11 (25.51–42.71)	
Autologous stem cell transplant				
No	31	9	46.70 (39.95–53.44)	0.397
Yes	41	8	50.94 (45.31–56.58)	
Response to ASCT, After 100 days				
No ASCT	31	9	46.70 (39.95–53.44)	0.672
Complete response	25	5	50.27 (42.71–57.84)	
VGPR & PR	16	3	47.75 (40.36–55.15)	
R-ISS				
Stage II	19	3	51.81 (44.29–59.32)	0.382
Stage III	53	14	48.32 (43.08–53.56)	
Maintenance treatment after ASCT				
No	12	4	39.32 (31.29–47.36)	0.484
Yes	60	13	50.08 (45.32–54.84)	
Consolidation Therapy				
LEN/DEX	18	2	49.85 (43.37–56.33)	0.893
KRD	8	2	48.86 (35.78–61.93)	
VRD	9	3	42.11 (30.41–53.81)	
KD	13	3	48.37 (38.11–58.63)	
POM/DEX	16	4	47.37 (37.40–57.34)	
VEL/DEXA	8	3	37.98 (28.38–47.58)	
Response to Consolidation Therapy				
Complete response	16	0	(1)	0.203
Very good partial response	14	3	48.71 (38.74–58.69)	
Partial response	21	4	49.85 (41.82–57.87)	
Stable disease	12	5	36.61 (27.89–45.33)	
Progressive disease	9	5	32.30 (22.43–42.18)	

VCD: Bortezomib–cyclophosphamide–dexamethasone therapy; ASCT: Autologous stem cell transplant; R-ISS: Revised International Staging System; LEN/DEX: Lenalidomide–dexamethasone; KRD: Carfilzomib–lenalidomide–dexamethasone; VRD: Bortezomib–lenalidomide–dexamethasone; KD: Carfilzomib–dexamethasone; POM/DEX: Pomalidomide plus low-dose dexamethasone; VEL/DEXA: Bortezomib–dexamethasone; CI: Confidence interval. (1) No statistics are computed because all cases are censored.

**Table 5:** Significant factors independently associated with mortality (multivariate cox proportional hazards model).

	β Coefficient	Standard Error	*p*	HR	95.0% CI for HR	
Second Primary malignancy	1.486	0.597	0.013	4.420	1.371	14.246
ALT	0.017	0.008	0.038	1.017	1.001	1.033

ALT: Alanine aminotransferase; CI: Confidence Interval, HR: Hazard ratio.

## Data Availability

The data that support the findings of this study are available from the corresponding author, upon reasonable request.

## Abbreviations

SPM: second primary malignancy; MM: multiple myeloma; ASCT: autologous stem cell transplants; IMIDs: immune modulatory agents; IMWG: International Myeloma Working Group; MDEs: myeloma-defining events; FLC: free light chain; R-ISS: International Staging System; BMI: body mass index; ADLs: activities of daily living; ECOG: Eastern Cooperative Oncology Group; LDH: lactate dehydrogenase; ALT: alanine aminotransferase; AST: aspartate aminotransferase; GGT: gamma-glutamyl transferase; eGFR: glomerular filtration rate; sFLC: serum-free light chain.
